# Supplementary material for: Evidence of Josephson Coupling in a Few-Layer Black Phosphorus Planar Josephson Junction
Source: ACS Nano. 2022 Jan 31;16(3):3538–45. doi: 10.1021/acsnano.1c09315 (PMC8945388; doi:10.1021/acsnano.1c09315)
Supplement: Supplementary file 1 — nn1c09315_si_001.pdf [file nn1c09315_si_001.pdf]

Supporting Information
Evidence of Josephson coupling in a few-layer black phosphorus planar Josephson junction

Francesca Telesio,[†] Matteo Carrega,[‡] Giulio Cappelli,[†] Andrea Iorio,[†] Alessandro Crippa,[†] Elia Strambini,[†] Francesco Giazotto,[†] Manuel Serrano–Ruiz,[¶] Maurizio Peruzzini,[¶] and Stefan Heun[*,†]

†*NEST, Istituto Nanoscienze-CNR and Scuola Normale Superiore, Piazza San Silvestro 12, 56127 Pisa, Italy*

‡*CNR-SPIN, Via Dodecaneso 33, 16146, Genova, Italy*

¶*CNR-ICCOM, Via Madonna del Piano 10, 50019 Sesto Fiorentino, Italy*

E-mail: stefan.heun@nano.cnr.it

Additional information on device fabrication

The bP crystals were prepared by heating commercially-available red phosphorus ($>$ 99.99%) in a muffle oven, together with Sn ($>$ 99.999%), Au ($>$ 99.99%), and a catalytic amount of SnI_4, following a published procedure.[1] The solids were loaded into a quartz tube, which was then evacuated by a pumping procedure: the vacuum was back-filled by N_2 gas several times, and then the tube was sealed under vacuum. Then it was heated up to 406 °C (at a rate of 4.2 °C/min), kept 2 hours at this temperature, and then heated up to 650 °C (2.2 °C/min). The sample was kept for three days at this temperature in the oven. Afterwards, a slow cooling rate was chosen (0.1 °C/min) to promote the formation of crystals of bP (typical size: 2 mm × 3 mm).

Raman spectroscopy

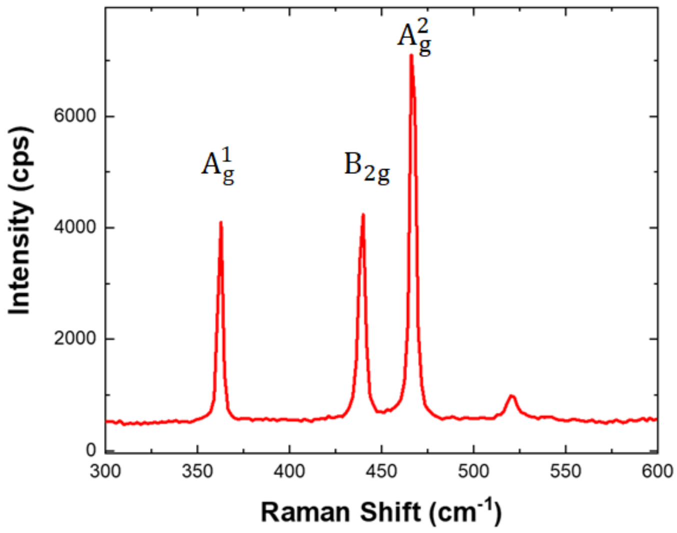

Figure S1: Typical Raman spectrum: the three characteristic Raman peaks of bP, the A_g^1, the B_{2g}, and the A_g^2 (labeled in the picture) are clearly visible, as well as the one related to the Si/SiO_2 substrate. The spectrum was acquired with a Renishaw inVia system equipped with a 532 nm laser.

Calculation of the McCumber parameter β_c

The capacitance of a parallel plate capacitor is $C = \epsilon_0 \epsilon_r \frac{A}{d}$, with A the height h of the superconducting lines (h = 10 nm Ti + 60 nm Nb = 70 nm) times the width W of the Josephson junction ($W = 2$ μm), and d the length L of the junction ($L = 500$ nm). With $\epsilon_r = 1$ for vacuum/air we get $C = 2.5 \times 10^{-18}$ F. The McCumber parameter is $\beta_c = \frac{2e}{\hbar} I_c R_N^2 C$. With $I_c = 5$ nA and $R_N = 560$ Ω we get $\beta_c = 1.2 \times 10^{-5} \ll 1$. We underline that this result will not significantly change even if we set $\epsilon_r \sim 4$ (for bP or SiO_2).

Additional transport characterization

Additional back gate sweeps

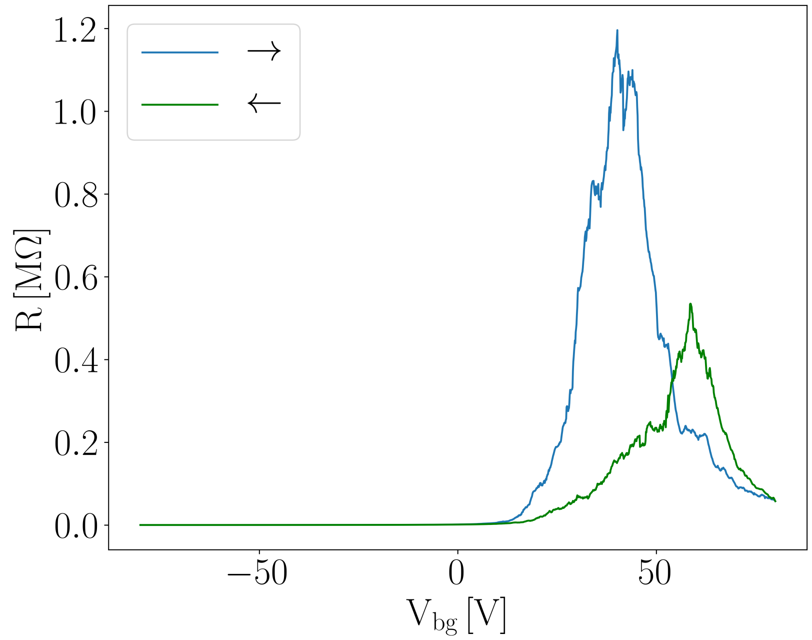

Figure S2: Resistance R *versus* back gate voltage V_{bg} for the −80 V to +80 V range, measured in current bias with a current of 90 nA. The device shows an ambipolar behavior and hysteresis in the depletion region. $B = 0$ mT, $T = 4.2$ K.

Additional $V - I$ curves

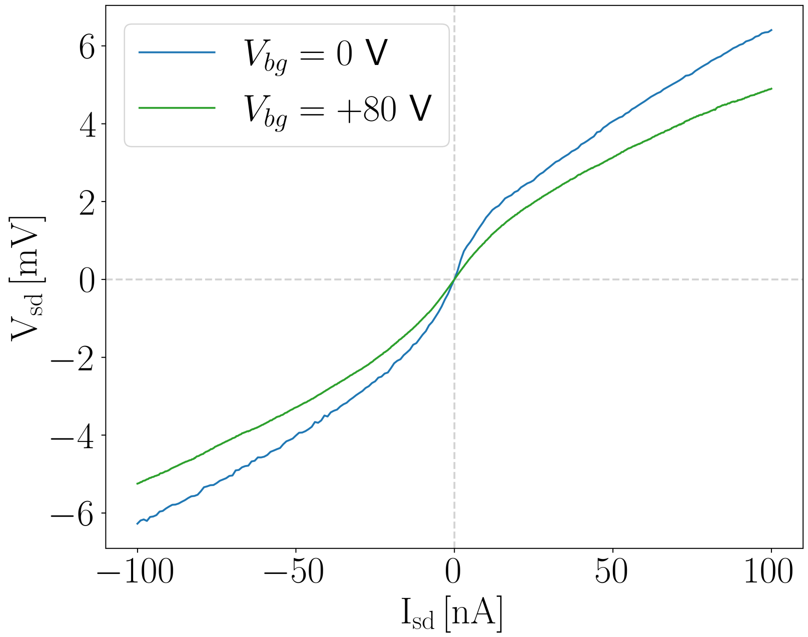

Figure S3: V_{sd} as a function of I_{sd} for back gate voltages $V_{bg} = 0$ V and $V_{bg} = +80$ V. $B = 0$ mT, $T = 2.3$ K.

As shown in Fig. S3, also in the n-type regime, at $V_{bg} = +80$ V, the quality of the contacts does not improve, consistent with previous observations,[2] and the metal-semiconductor contacts are still dominated by a Schottky barrier, which prevents observation of a bipolar signal in supercurrent amplitude.

We inspect the behavior of the junctions at elevated bias $V_{sd} \sim 2\Delta_S$, to look for any subharmonic gap features. Figure S4(a) shows a $V - I$ curve measured over a large bias range of $I_{sd} = \pm 5$ μA, at back gate voltage $V_{bg} = -80$ V. As shown in Fig. S4(b), a linear fit to the part with $V_{sd} > 1.5$ mV yields no excess current, but rather a small deficit current of -40 nA, indicated by the arrow. The situation remains like this also for smaller negative voltages on the back gate, with a deficit current observed for all back gate voltages, which increases to -200 nA for $V_{bg} = -40$ V. This points at the interface transparency as an important (limiting) factor, suggesting the possibility of a barrier between the S and N parts of the junction, so that the junction would be better described as a SINIS structure, *i.e.*, a

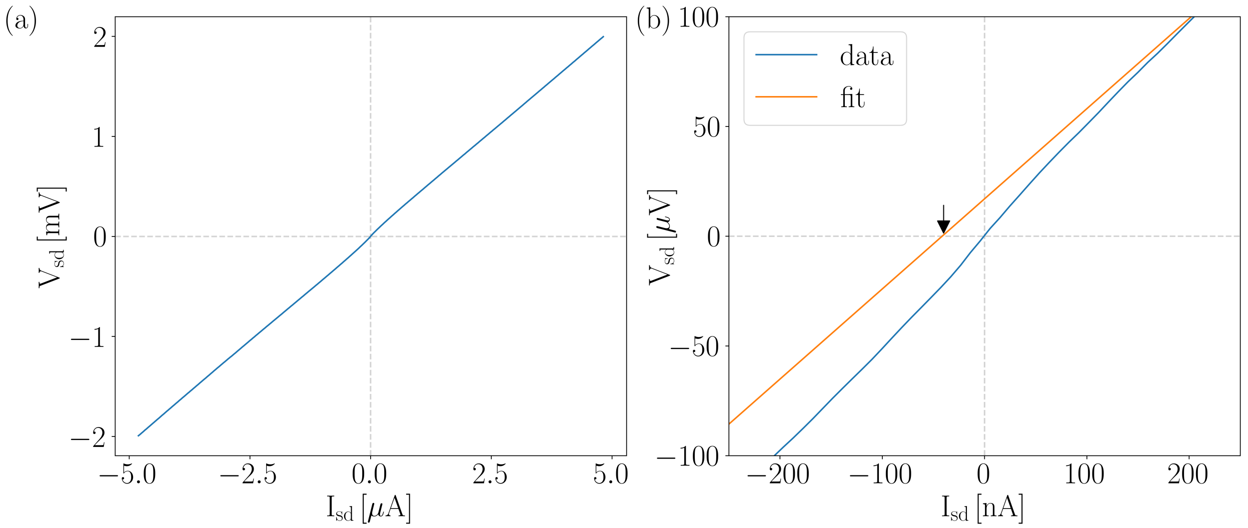

Figure S4: (a) V_{sd} as a function of I_{sd} for a wider range of bias values, at back gate voltage −80 V. (b) Zoom-in to the central part of the curve. The linear fit to the part of the curve with $V_{sd} > 1.5$ mV shows a deficit current of −40 nA, indicated by the arrow. $B = 0$ mT, $T = 33$ mK.

superconductor-insulator-normal-insulator-superconductor hybrid system.

These conclusions are consistent with the simultaneously measured differential resistance curves. One example, measured at $V_{bg} = -80$ V, is shown in Fig. 1(d) of the main text. The curve does not show any evidence for multiple Andreev reflections (MARs), but instead a clear Schottky behavior, with an increased resistance at the origin. From the ratio in differential resistance at zero bias and at high bias, we estimate that the resistance of the S-N interfaces contributes about 30% to the overall resistance of the junction.

Data from device $1-2$

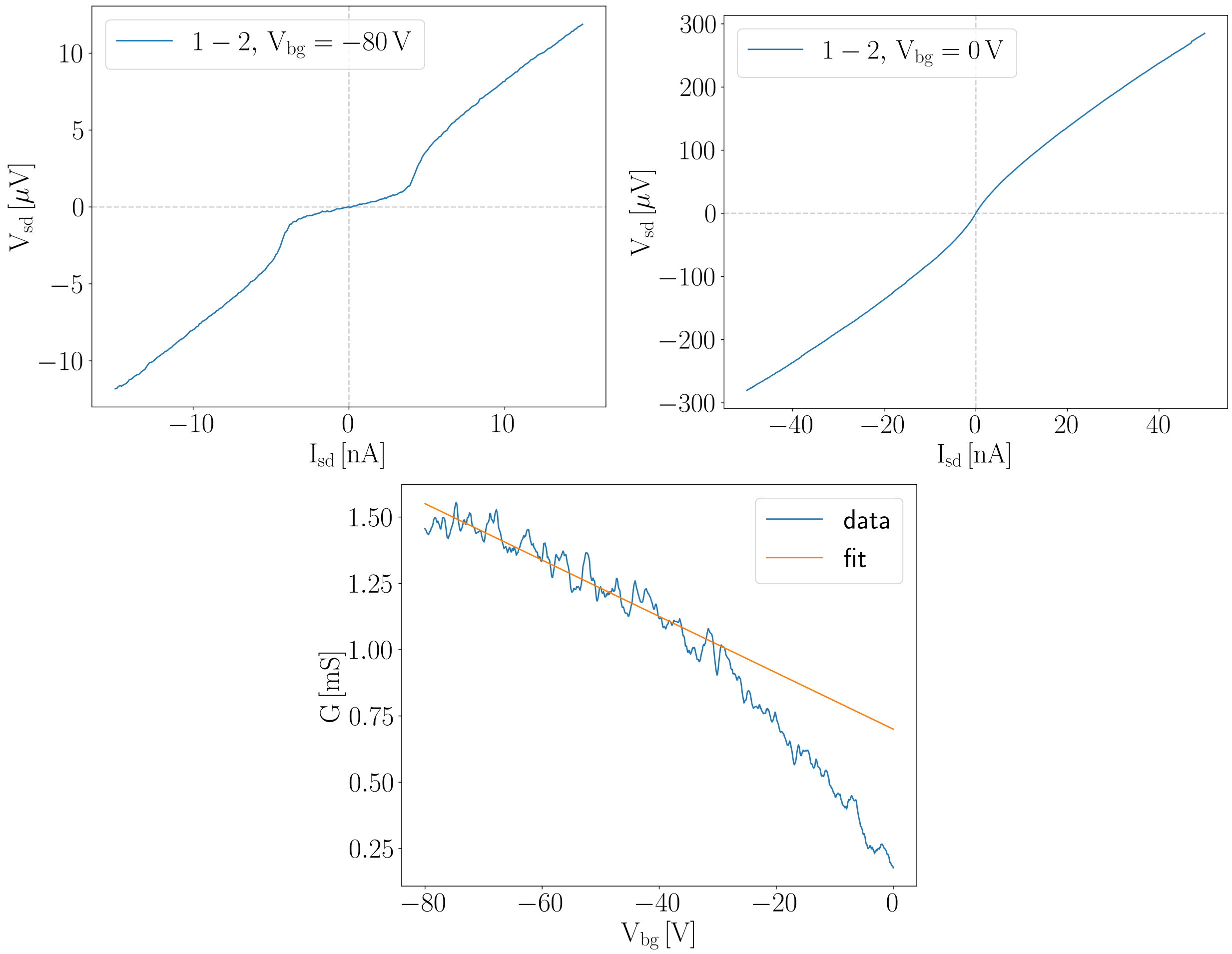

Figure S5: (a) V_{sd} as a function of I_{sd} at a back gate voltage $V_{bg} = -80$ V. (b) Same for back gate voltage 0 V. (c) Conductance G *versus* back gate voltage V_{bg} from -80 V to 0 V, measured in current bias with a current of 50 nA. The straight line is a linear fit to the data from $V_{bg} = -80$ V to -30 V, used to calculate the hole mobility. A field effect mobility $\mu = 231$ cm^2/(Vs) is obtained. Data measured at $B = 0$ mT and $T = 33$ mK.

Arrhenius plot

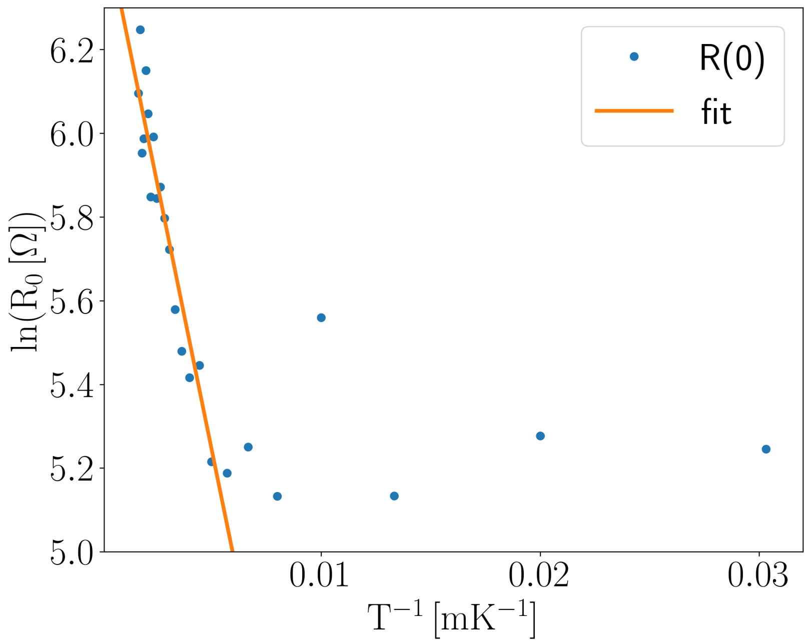

Figure S6: Arrhenius plot of $\ln(R_0)$ *versus* $1/T$. Between 200 mK and 600 mK the data in the log-plot approximately follow a linear trend, with slope -256.0 mK. From the slope, $E_J = 11$ μeV is obtained, in good agreement with the value of $E_J = 10.3$ μeV obtained at base temperature (33 mK). $B = 0$ mT, $V_{bg} = -80$ V.

References

(1) Nilges, T.; Kersting, M.; Pfeifer, T. A Fast Low-Pressure Transport Route to Large Black Phosphorus Single Crystals. *J. Solid State Chem.* **2008**, *181*, 1707–1711.

(2) Telesio, F.; le Gal, G.; Serrano-Ruiz, M.; Prescimone, F.; Toffanin, S.; Peruzzini, M.; Heun, S. Ohmic Contact Engineering in Few–Layer Black Phosphorus: Approaching the Quantum Limit. *Nanotechnology* **2020**, *31*, 334002.
